# Conditional Random Fields for Fast, Large-Scale Genome-Wide Association Studies

**DOI:** 10.1371/journal.pone.0021591

**Published:** 2011-07-12

**Authors:** Jim C. Huang, Christopher Meek, Carl Kadie, David Heckerman

**Affiliations:** Microsoft Research, Redmond, Washington, United States of America; University of Texas, United States of America

## Abstract

Understanding the role of genetic variation in human diseases remains an important problem to be solved in genomics. An important component of such variation consist of variations at single sites in DNA, or single nucleotide polymorphisms (SNPs). Typically, the problem of associating particular SNPs to phenotypes has been confounded by hidden factors such as the presence of population structure, family structure or cryptic relatedness in the sample of individuals being analyzed. Such confounding factors lead to a large number of spurious associations and missed associations. Various statistical methods have been proposed to account for such confounding factors such as linear mixed-effect models (LMMs) or methods that adjust data based on a principal components analysis (PCA), but these methods either suffer from low power or cease to be tractable for larger numbers of individuals in the sample. Here we present a statistical model for conducting genome-wide association studies (GWAS) that accounts for such confounding factors. Our method scales in runtime quadratic in the number of individuals being studied with only a modest loss in statistical power as compared to LMM-based and PCA-based methods when testing on synthetic data that was generated from a generalized LMM. Applying our method to both real and synthetic human genotype/phenotype data, we demonstrate the ability of our model to correct for confounding factors while requiring significantly less runtime relative to LMMs. We have implemented methods for fitting these models, which are available at http://www.microsoft.com/science.

## Introduction

Population structure, family structure and/or cryptic relatedness are well-known confounding factors that cause spurious associations to be found in GWAS [1]–[6]. Standard statistical hypothesis testing of association between markers and phenotypes can produce a large number of false positive associations, as SNP markers may be correlated with phenotype purely as a result of confounding factor effects. As the cost of genotyping drops and the sizes of such studies continue to grow above tens of thousands of individuals [7]–[9], the influence of such confounding effects on GWAS will become more acute, requiring statistical analysis methods that will both scale for large numbers of individuals while accounting for the confounders.

The standard techniques for dealing with confounding factors fall into several classes. An effective class of methods includes approaches formulated as LMMs [10], which model confounding factors using pairwise similarity measures between every pair of individuals. As the effects of confounders are all encoded in the set of SNPs carried by all individuals, the set of similarities can then be used in a regression model to distinguish between spurious and true SNP-phenotype associations. Other methods have been proposed that use a principal components analysis of individuals' SNPs [4], perform a post-hoc correction of test statistics such as Genomic Control [2], or cluster individuals before performing an aggregate association between clusters and phenotypes [11]. These methods, while accounting for confounding factors under different assumptions, have been shown to either suffer from insufficient statistical power when the confounding effects are strong [4], [5] or are unable to fully capture their effects altogether, such that many false positives are produced [3], [5], [12]. In several recent studies [3], [5], [12], [13], methods based on LMMs were found to produce fewer false positives and had higher statistical power as compared to other methods for modeling confounding factors, making LMMs a popular class of GWAS methods that have high statistical power and low false positive rates.

Although LMMs have been shown to effectively model and correct for confounding factors in GWAS, an important problem that remains to be solved is how to minimize the computational costs of such methods. Methods based on LMMs typically incur high computational costs, particularly for studies with larger numbers of individuals, as the matrix operations required for parameter estimation scale cubically with the number of individuals. In the regime where the number of individuals grows large and where confounding factors exert strong effects, this may hinder the applicability of LMMs. One possible approach to the above problem is to turn to alternative classes of models that allow us to model similarities between individuals in order to account for confounding factors in the data (as do LMMs) while eschewing the need for costly matrix operations during parameter estimation. In particular, probabilistic graphical models are a natural class of statistical models that allow both for modeling similarities between individuals and fast parameter estimation. In this paper we propose a probabilistic graphical model and parameter estimation method for associating SNPs to phenotype that both accounts for confounding factors and runs significantly faster than current LMM-based methods for larger numbers of individuals, allowing the method to scale to larger study sizes. Unlike LMM-based methods (which present local optima in parameter estimation [3]) or PCA-based methods [4], our method for parameter estimation is not prone to local optima and is also guaranteed to yield unique, globally optimal parameter estimates. We will apply our model to real and synthetic human genotype datasets, where we show significantly lower runtimes for our method as compared to LMM-based methods for larger study sizes, with only a modest loss in statistical power relative to LMM-based methods when testing on synthetic data that was generated from a generalized LMM. Finally, we have implemented methods for fitting these models, which are available at http://www.microsoft.com/science.

## Results

**Figure 1:**
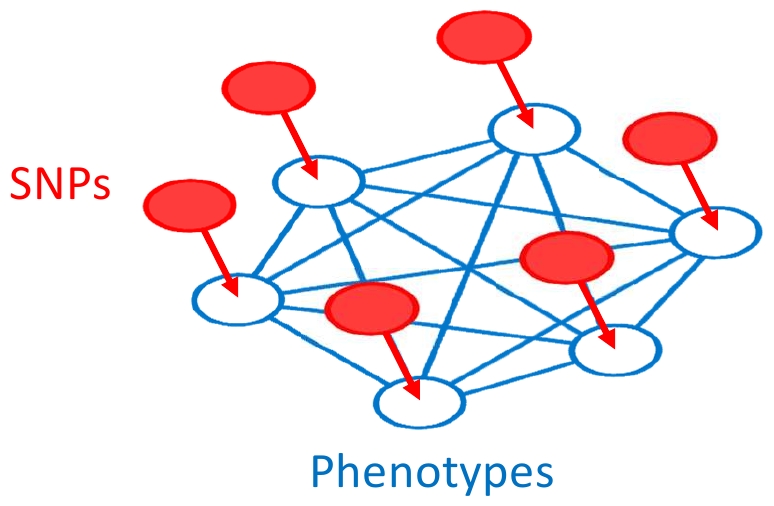
The graphical model for relating genetic variation to phenotype. Nodes correspond to variables in the model and edges correspond to dependencies between variables under the model. Shaded nodes correspond to observed variables under the model. Conditioned on each individual's SNP and covariates, phenotypic labels are modeled using a fully connected undirected graphical model.

We present a model for relating individuals' phenotypic labels as a function of a given SNP marker and other covariates. The output of our model will be some statistic for the SNP marker, so that we can perform a GWAS by applying our model to each SNP marker in a large set of interest. Given a set of individuals, we assume that phenotypes consist of binary labels corresponding to the absence/presence of a phenotype in an individual, although the model can easily be generalized to polytomous discrete or continuous phenotypes. For a given locus, our model specifies a joint probability over individuals' observed phenotypes, conditioned on each individual's SNP and covariates. The joint probability will be a function of all pairs of individuals' phenotypes and each individual's SNP and covariates. Under our model, the contribution of each pair of individual phenotypes will increase or decrease as a function of the genetic similarity between the pair of individuals. Analogously, the contribution of each individual's SNP and covariates will vary as a function of how strongly the SNP and covariates influence that individual's phenotype, taking into account genetic similarity between individuals. The dependencies between individuals due to genetic similarity, in addition to the influence of genetic variation and covariates in generating phenotypes, can be modelled using a graph in which nodes correspond to observed phenotypes and covariates. Edges in the graph denote dependencies between phenotypes and covariates (Figure 1).

The goal of associating SNPs to phenotypes then corresponds to parameter estimation under our model in which genetic similarity between individuals is accounted for (see Methods for more details). For a given SNP, the model parameters can be assigned a p-value which we will use as a test statistic of significance of association between the given SNP and individuals' phenotypes under the null hypothesis that no associations hold between genetic variation and phenotype (see Methods). To test the utility of the proposed model for association studies, we describe in the next section a series of experiments that measure the degree to which the above model accounts for confounding factors and its computational cost for larger studies.

### Experiments

Given our probabilistic model for estimating associations between SNPs and phenotype, we would like to test two aspects of the model. The first is that of *calibration*, or whether the distribution of p-values is uniform under the null hypothesis for each SNP. On synthetic data, it is straightforward to guarantee this condition. On real data, we use our prior belief that very few SNPs are associated with the phenotype to obtain this condition. As is standard practice in GWAS, we summarize the departure of an observed p-value distribution from the theoretical null distribution by use of the 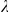 statistic, or genomic inflation factor [2], which measures how much smaller the observed median p-value is compared to that expected in the theoretical null distribution. Therefore, on data containing no (or very few) associations, 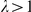 suggests that the p-value distribution is inflated (too many small, significant p-values), which can happen when confounding factors are inadequately modeled. Conversely, 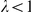 implies deflated p-values (too few small p-values). In general, small variations from 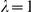 are expected to occur even in synthetically generated datasets with no associations due to sampling error for a finite number of SNPs.

The second aspect we wish to test is that of *discrimination*, or whether the model can distinguish spurious associations from real ones. To do this, we must apply our method to data where the ground truth as to the strength of associations to be found is known at the outset. Ideally, we would sample individuals' phenotypes under the undirected graphical model, conditioned on their SNPs and covariates. However, obtaining samples from the correct joint probability is in general intractable (see Methods). An alternative is to instead generate synthetic data from a generalized linear mixed model (GLMM), which is tractable, and then assess the statistical power of our method in distinguishing between spurious and true associations for this dataset (see Methods for details on how synthetic data were generated). One caveat is that sampling from the GLMM would mean that our model is misspecified and would suffer some loss of power relative to a LMM when both are applied to the sampled data. However, provided that we are able to generate data similar to real genotype/phenotype data, the analysis on synthetic data will inform us about whether the method will have significant statistical power on real data. Furthermore, if the outputs of our model are similar between synthetic and real data, then this would suggest that the model has adequately captured the statistics of the data in the sense of modeling confounding factors.

To test the above two aspects, we used both real data and synthetic phenotypes generated from a GLMM using real human genotype and phenotype data. The GAW14 [14] dataset consists of 7,579 SNP markers for 1,261 individuals from four distinct subpopulations (white non-Hispanic, black non-Hispanic, Hispanic, and other), where an individual's phenotype corresponds to whether he/she smokes or not. We also used the GOLDN dataset [15] which consisted of 647 SNP markers for 1,114 individuals from two National Heart, Lung and Blood Institute (NHLBI) Family Heart Study (FHS) field centers, where an individual's phenotype corresponds to whether he/she is above or below the population median height. In both datasets, due to a large amount of population structure and family structure, it is expected that the effects of confounding factors will be strong.

**Figure 2:**
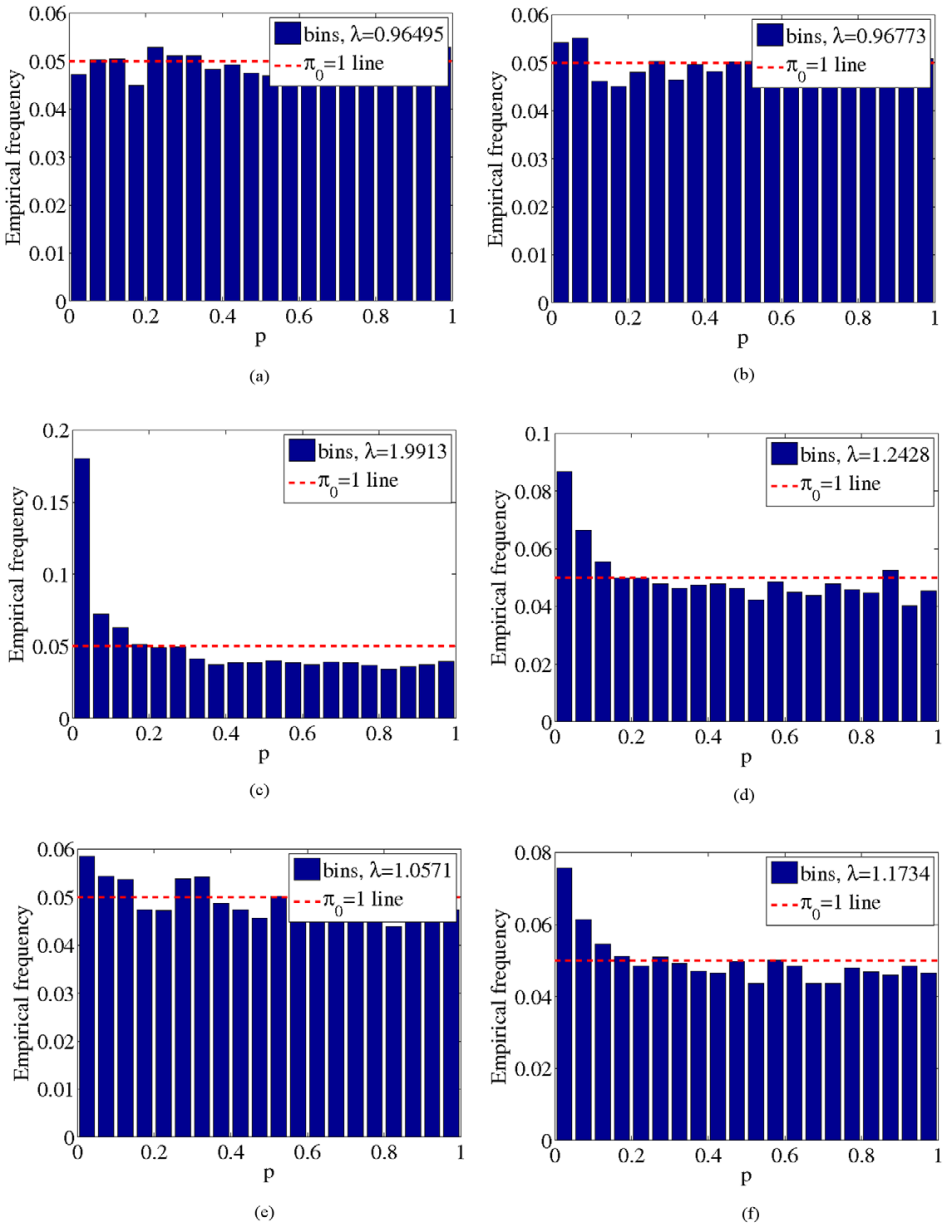
P-value histograms for the GAW14 dataset. a),b) Histograms of p-values obtained from our method for the synthetic (a) and real (b) GAW14 data. For comparison, p-values obtained from a logistic regression that does not account for confounding factors and from Eigenstrat [4] are shown for synthetic (c,e) and real (d,f) GAW14 data. Dotted red lines indicate the expected histogram for the uniform distribution under the null hypothesis 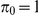.

**Figure 3:**
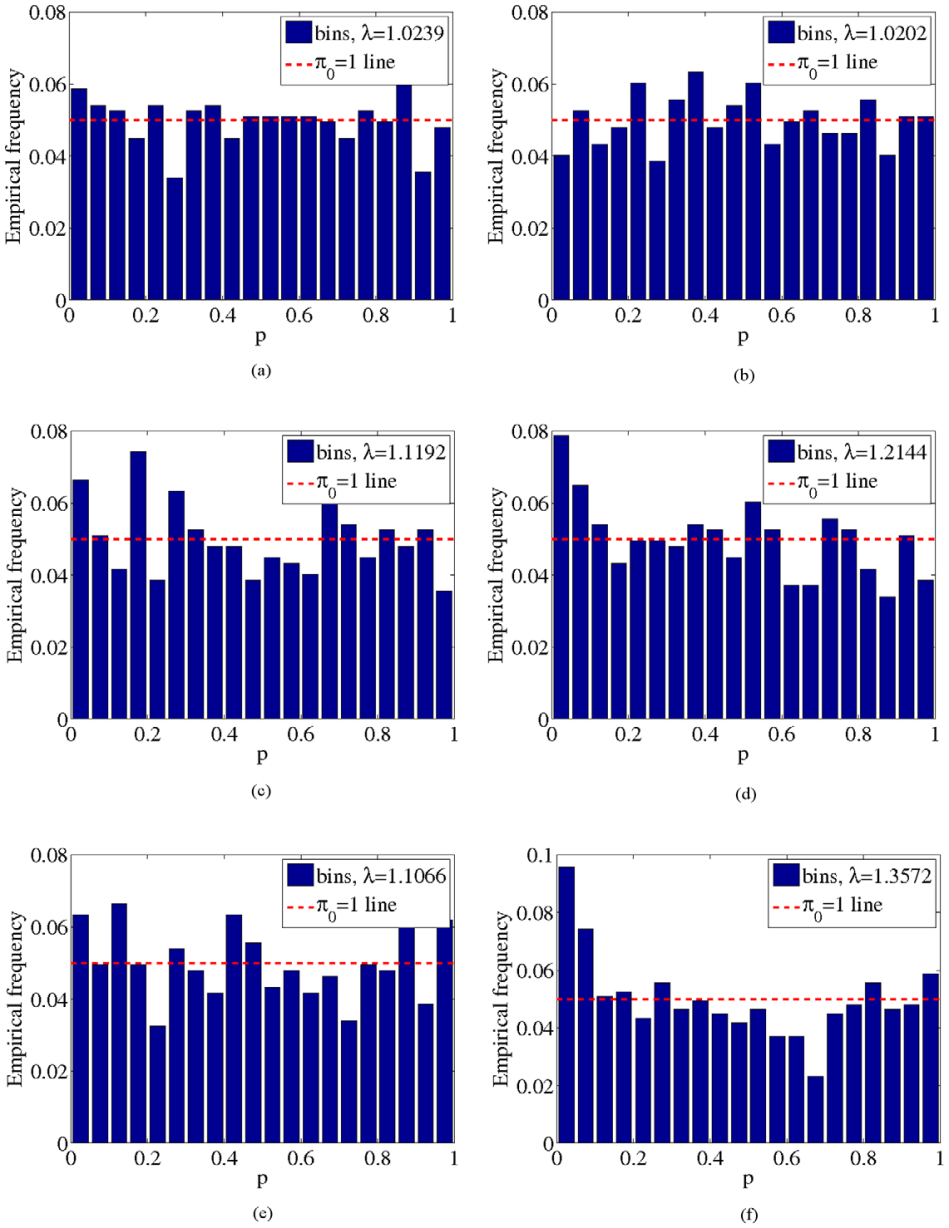
P-value histograms for the GOLDN data. a),b) Histograms of p-values obtained from our method for the synthetic (a) and real (b) GOLDN data. For comparison, p-values obtained from a logistic regression that does not account for confounding factors and from Eigenstrat [4] are shown for synthetic (c,e) and real (d,f) GOLDN data. Dotted red lines indicate the expected histogram for the uniform distribution under the null hypothesis 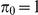.

**Figure 4:**
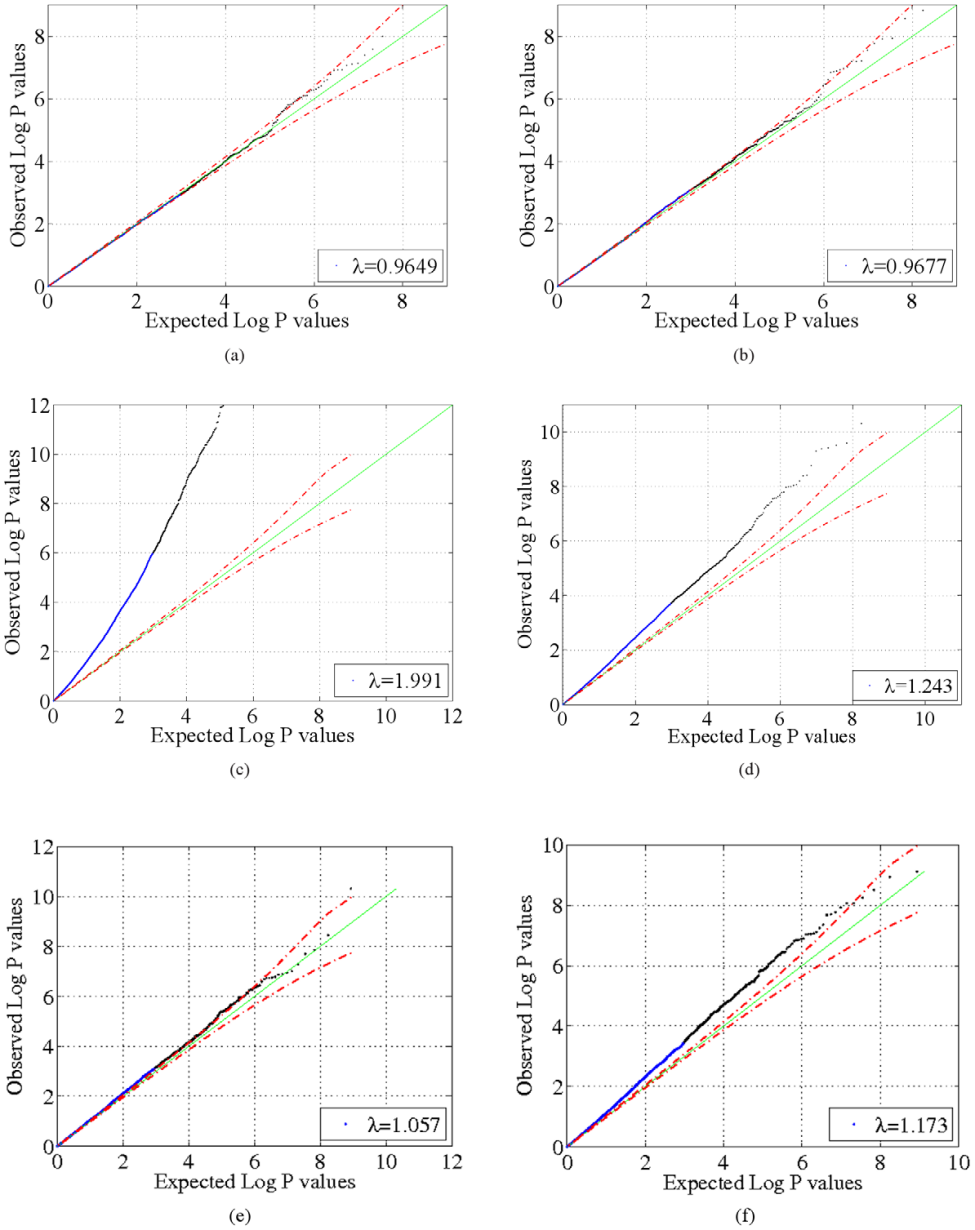
Quantile-quantile (QQ) plots for the GAW14 data. QQ plots of model negative 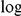 p-value statistics obtained from our method as a function of expected negative 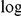 p-values under the null hypothesis 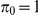 for the synthetic GAW14 data with 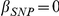 (a) and real data (b). For comparison, negative 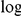 p-value statistics obtained from a logistic regression that does not account for confounding factors and from Eigenstrat [4] are shown for the synthetic data with 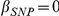 (c,e) and real data (d,f). Dotted red lines indicate 95% confidence bounds.

**Figure 5:**
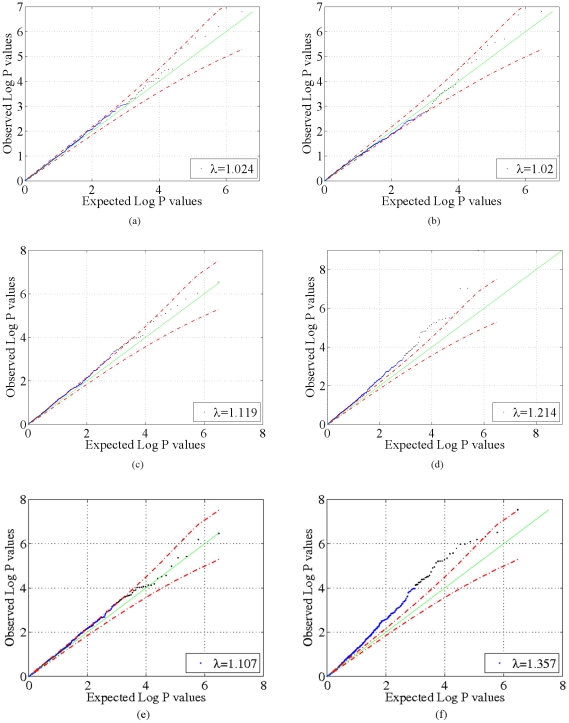
Quantile-quantile (QQ) plots for the GOLDN data. QQ plots of model negative 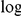 p-value statistics obtained from our method as a function of expected negative 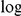 p-values under the null hypothesis 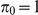 for synthetic GOLDN data with 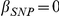 (a) and real data (b). For comparison, negative 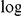 p-value statistics obtained from a logistic regression that does not account for confounding factors and from Eigenstrat [4] are shown for the synthetic data with 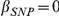 (c,e) and real data (d,f). Dotted red lines indicate 95% confidence bounds.

**Figure 6:**
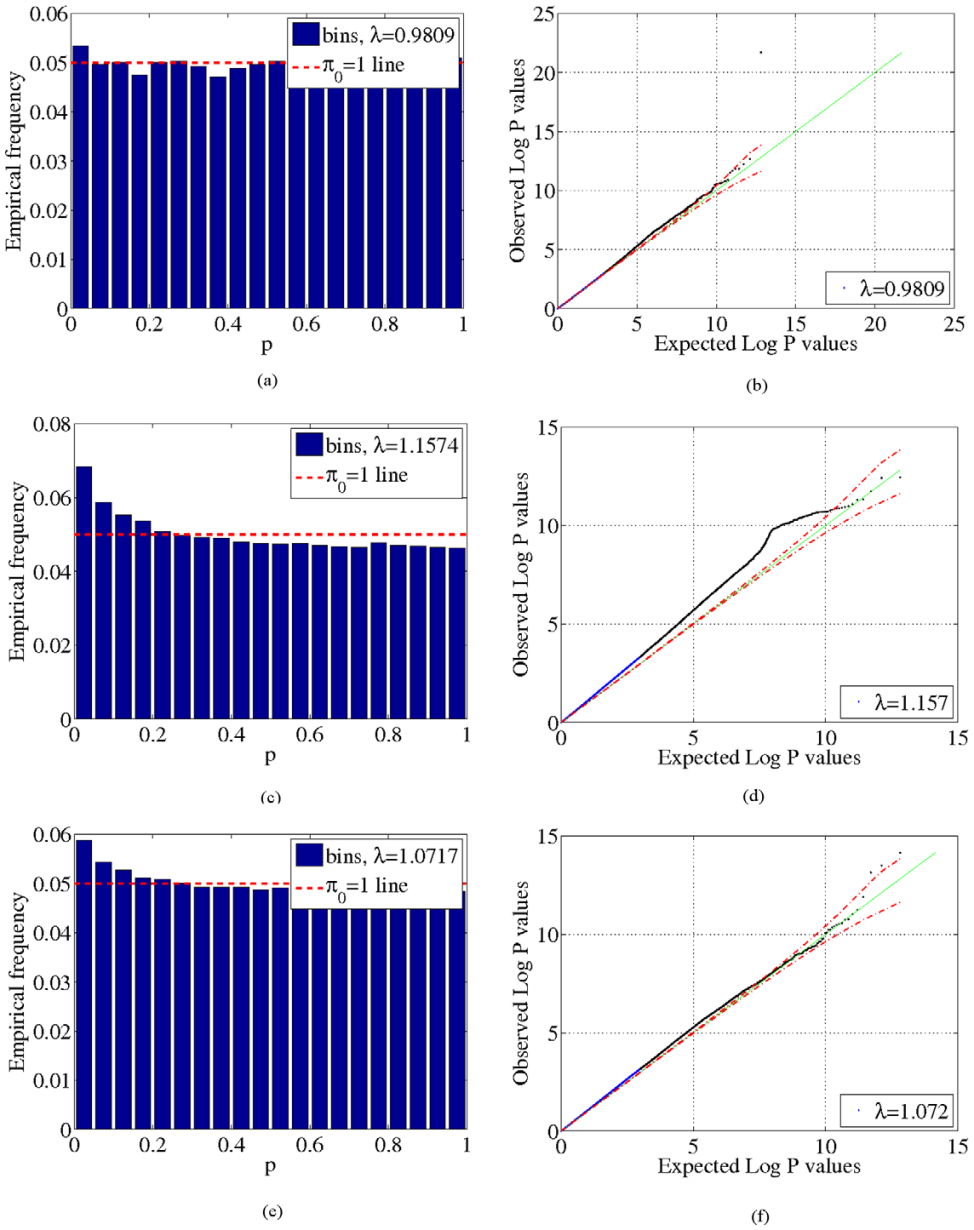
Histograms and quantile-quantile (QQ) plots for the WTCCC data. Negative 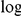 p-value statistics obtained from our method (a,b), logistic regression that does not account for confounding factors (c,d) and from Eigenstrat [4] (e,f) for the WTCCC data. Dotted red lines in the QQ plots (right panel) indicate 95% confidence bounds.

We applied our model to the above real datasets and to the synthetically-generated data, where for all datasets, individual age covariates were binned into five ranges 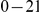, 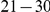, 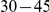, 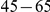, 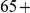 and each individual's age group, encoded as a binary 5-vector, was used in the regression. All covariates and SNP values were standardized to have zero mean and unit standard deviation across individuals. We see that the distributions of p-values in both real and synthetic datasets are not significantly different from the uniform distribution of p-values that is expected under the null hypothesis, as measured by both one-sample Kolmogorov-Smirnov tests (p = 0.16,0.13 for the synthetic and real GAW14 data, p = 0.74,0.74 for synthetic and real GOLDN data) and the genomic inflation factor 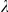, shown in Figures 2(a,b), 3(a,b), 4(a,b) and 5(a,b). These two results suggest that our model adequately models confounding factors and has a low false positive rate in the presence of confounders. For comparison, Figures 2(c,d,e,f), 3(c,d,e,f), 4(c,d,e,f) and 5(c,d,e,f) show p-values obtained from 1) a logistic regression of phenotype onto covariates and SNPs without accounting for confounding factors and 2) from using the PCA-based Eigenstrat method [4]. Here we see that an inflation of the number of significant p-values occurs for these latter two methods, as the distribution of p-values obtained deviates significantly from the uniform distribution (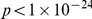). One possible explanation for the inflation seen in the p-values produced by the PCA-based method is that it may be biased against due to the relatively small number of markers evaluated in the GAW14 and GOLDN datasets. However, upon additional evaluations on the larger Wellcome Trust Case Control Consortium dataset [16] (Figure 6) containing of 360,657 SNP markers across 3,400 individuals, we observe similar results in that the PCA-based method again produces inflated p-values, whereas our method produced no significant deviation from the uniform distribution of p-values expected under the null hypothesis. We also note that the distributions of p-values obtained are similar for both real data and synthetic data in which the SNP regression weight is set to 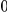 (Figures 2,3), suggesting that our sampling method has produced synthetic data which is representative of real data.

**Figure 7:**
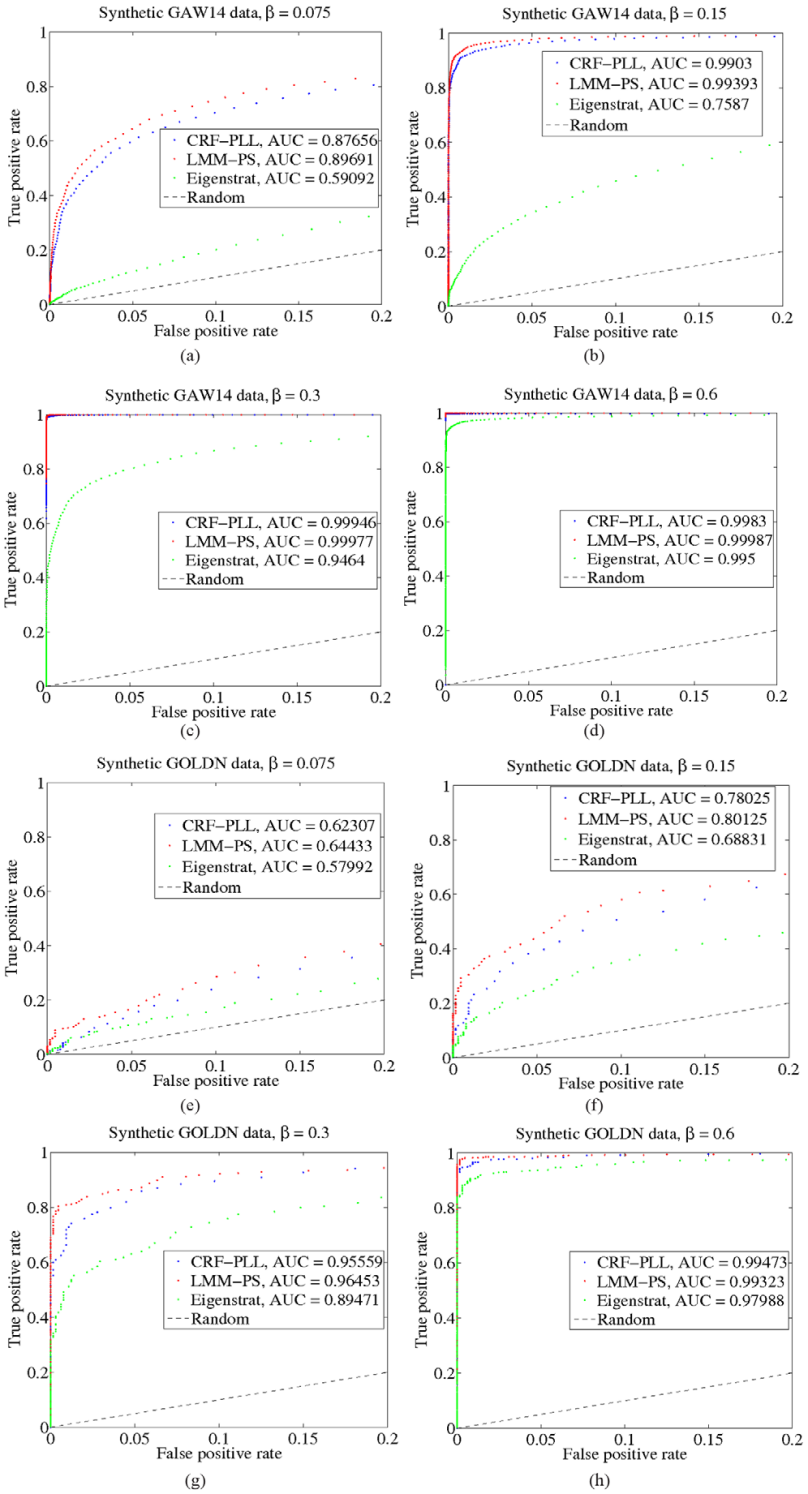
Assessing statistical power on synthetic data. The plots are shown as receiver operating characteristic (ROC) curves of the true positive rate as a function of the false positive rate for the GAW14 dataset (a,b,c,d) and the GOLDN dataset (e,f,g,h) for various values of the SNP regression weight when using our method (blue), a LMM-based method (red), a PCA-based method (green) and random guessing (black dotted).

In addition to testing the calibration of our method, we would also like to test its ability to distinguish spurious associations from real ones, or its statistical power. A method that produces few significant p-values for data where 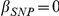 and many significant p-values for data where 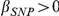 will have high statistical power, as measured by true and false positive rates. The results of the synthetic experiments are shown in Figure 7 for the GAW14 and GOLDN datasets. The plots are shown as receiver operating characteristic (ROC) curves of the true positive rate as a function of the false positive rate (see Methods). The performance of our model can then be summarized using the area under the ROC curve, or AUC, which is high if our model has high statistical power in discriminating between real and spurious associations. For comparison, we also applied the LMM-based method of [12], which also accounts for confounding factors, to the above synthetic data using the same set of similarities as that used by our method, but interpreted instead as a covariance matrix among individuals under a multivariate Gaussian distribution. As an additional point of comparison, we also applied the Eigenstrat method [4] to the synthetic data. As expected, due to the mismatch between the model used to generate the synthetic test data and our model, there is a modest loss in power as compared to the LMM, whereby the loss in model power decreases as the SNP weight 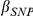 is increased (Figure 7). The loss in power is partially explained by noting that the data was generated from a GLMM using a Gaussian covariance matrix 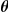, which corresponds to the same covariance matrix used in the LMM. However, 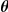 in our model cannot be interpreted as a covariance matrix under a multivariate Gaussian distribution, implying a larger mismatch between our model and the data as compared to that between the LMM and the data. We also see that Eigenstrat, while having low computational cost, does not adequately account for confounding factors and so has significantly lower power as compared to our method.

**Figure 8:**
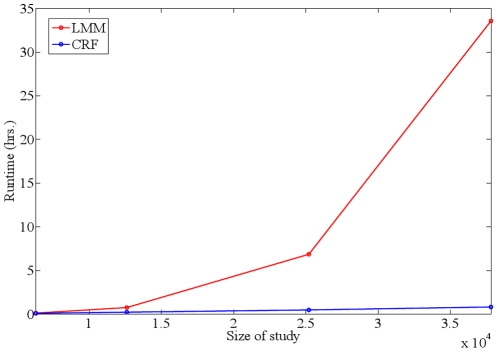
Comparison of runtimes. Runtimes for the CRF and LMM models (in hours) are shown as a function of study size. All experiments were run on a machine with two 3.0 GHz CPUs and 64.0 GB of RAM.

In addition to assessing the statistical power of our method, we also assessed the runtime of our method as a function of the study size, or number 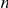 of individuals. To do this, we synthesized datasets consisting of the phenotypes, SNPs and similarities of the GAW14 dataset replicated several times (up to 35,000 individuals), such that each synthetic dataset generated this way has an increasing number of individuals. We then applied both our method and the LMM to the synthetic datasets and recorded the total time taken to perform a GWAS for each dataset. All experiments were run on a single machine running Windows Server Enterprise with two Intel Xeon E5450 3.0 GHz 64-bit CPUs with 64.0 GB of RAM. Figure 8 shows the runtime of both methods as a function of the study size: as can be seen, the runtime for estimating the parameters of the LMM grows quickly as the number of individuals increases, whereas for our method, the runtime does not grow quickly. In particular, the difference in runtime becomes acute as the study size exceeds 20,000 individuals, resulting in significant runtime speedups (48 mins. for our method as compared to over 33 hours for the LMM for a study with 37,830 individuals). We remark here that although the experiments were carried out on a single machine, the differences in runtime of our method over the LMM-based method would also apply for experiments carried out on computation clusters with multiple compute nodes.

## Discussion

We have presented a novel GWAS method that accounts for confounding factors such as population structure, family structure or cryptic relatedness. Similar to LMMs and PCA-based methods for association, our model accounts for confounding factors through the use of pairwise similarities between patients, which allows us to significantly reduce false positive rates when performing associations. In contrast to LMM-based and PCA-based methods, our method retains high statistical power and is relatively inexpensive even as the number of individuals in a study grows. Our experimental results on both real and synthetic genotype data demonstrate that our method can adequately account for confounding factors in order to reduce false positive rates, with a modest loss in statistical power as compared to LMM-based and PCA-based methods for data that is generated from a generalized LMM. We have shown that our method is significantly faster than methods based on LMMs, where significant speedups are obtained as the number of individuals in a study grows. As future studies grow to encompass tens of thousands of individuals [7]–[9], the speedups afforded by our method over LMM-based methods are expected to be even larger than ones shown here. Although other methods that also have fast runtimes for large datasets could be used, in the regime where the effect of confounders is even stronger than it is for smaller studies, it is expected that these methods will not be able to model confounders adequately so as to reduce false positive associations. Our method presents a reasonable tradeoff between statistical power, low false positive rates and runtime that make it ideally suited for application to larger association studies where other methods either produce too many false positives or incur high computational costs. Future work would involve extending the method to multinomial discrete phenotypes and for modeling multiple phenotypes simultaneously, examining the use of other pairwise similarity measures, or the possibility of incorporating additional covariates into the similarity measures themselves.

## Methods

### Datasets

#### GAW14 dataset

The GAW14 dataset consisted of a subset of the data provided to the Genetic Analysis Workshop 14 (GAW 14) as part of the Collaborative Study on the Genetics of Alcoholism (U10 AA008401), which is described in detail elsewhere [14]. A total of 1,279 individuals genotyped at 7,579 loci were used from the GAW14 dataset for our analysis. Genotypes are coded using the number of minor alleles,such that the SNP value at a given locus takes on values 0,1,2. Age, sex and ethnic sub-population were recorded for each individual and used as covariates in our analysis. Measured phenotypes included alcohol dependence and smoking activity: the smoking activity phenotype was used for our analysis.

#### GOLDN dataset

Details about the GOLDN study has been described in detail elsewhere [15]. Briefly, the largest three-generation families were recruited from the pool of families that had participated in the National Heart, Lung, and Blood Institute Family Heart Study (FHS) at either the Minnesota or Utah field centers. A total of 1114 individuals from 190 families, genotyped at 647 SNP markers, were included in our analysis. Genotype data was encoded as for the GAW14 dataset. Age and sex was recorded for each individual and used as covariates in our analysis. Measured phenotypes in this dataset included height, physical activity and cholesterol levels: the height phenotype was the one used for our analysis.

### WTCCC dataset

The Wellcome Trust Case Control Consortium (WTCCC) data consisted of SNP data for about 1,900 individuals with Crohn's disease and about 1,500 controls from the UK Blood Service Control Group (NBS). SNPs were excluded from analysis using the more conservative SNP filter described by the WTCCC in [16], wherein a SNP was excluded if either its minor-allele frequency less than 0.01, it was missing in greater than one percent of individuals, or it was in the extended MHC region. After filtering, 360,657 SNPs remained. Non-white individuals and close family members were not excluded.

### Genome-wide association studies using conditional random fields

Given a set of individuals 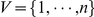, we assume that phenotypes consist of binary labels 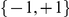 corresponding to the absence/presence of a phenotype in an individual, although the model can easily be generalized to polytomous discrete or continuous phenotypes. Denote by 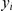 the observed phenotype for the 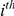 individual 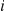 and let 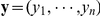 be the vector of observed phenotypes for all individuals in the study. Let 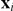 be the vector of covariates for individual 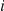 and let 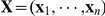 denote the matrix of covariates for the individuals in the study. Here, the covariates for an individual would include that individual's SNP marker at a given loci and possibly labels for age, gender and ethnicity.

For a given locus, our model consists of a probabilistic graphical model over individuals' observed phenotypes, conditioned on each individual's SNP and covariates. A probabilistic graphical model consists of two parts: the first is an graph 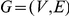 in which nodes in 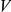 correspond to individuals and undirected edges in 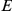 between pairs of nodes correspond to possible dependencies between the phenotypes of pairs of individuals. The second part of the model is a joint probability distribution on individuals' phenotypes that is a function of all pairs of individuals' phenotypes and each individual's SNP and covariates. Given a graph and the corresponding joint probability distribution, the graphical model captures both the dependencies between individuals due to genetic similarity, in addition to the influence of genetic variation and covariates in generating phenotypes. The influence of genetic variation and covariates is captured using a set of weights 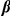, where a larger weight magnitude for a given covariate denotes an increased influence of that covariate on determining phenotype. The joint probability of phenotypic labels, conditioned on each individual's genetic variant and covariates is then given by
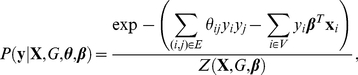
(1)where 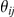 is a real-valued genetic similarity for edge 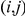 that models genetic similarity between individuals 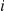 and 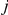, and 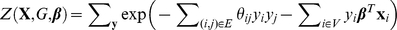 is the partition function that ensures that the probability sums to unity. In the above model, we assume that genetic similarities, denoted collectively as 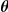, are provided and fixed. Various ways of setting the similarities can be used. Based on their previous use in LMMs [12], we found that using similarities based on Identity-by-State (IBS) worked best, where the IBS value between two individuals is equal to the fraction of SNP marker alleles that are shared between individuals [17] across the entire set of SNPs being studied. The use of the IBS similarity measure here allows us to account for the effects of confounding factors which are encoded in the set of SNPs carried by all individuals.

Given individuals' phenotypes 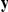 and covariates 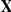 and a matrix of genetic similarities 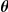, the goal is to estimate the effect of a particular SNP on the individuals' phenotypes by estimating the weight vector 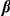. A common criterion that is used consists of maximizing the above probability with respect to the weights for the observed data, or the maximum-likelihood criterion. However, a key difficulty with the above model is that estimating the weights requires that we compute the partition function and its derivatives, which, for even a moderate number of individuals, will be intractable, as it requires summing over all possible joint configurations of the binary vector 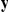. An alternative criterion for parameter estimation that does not require computing 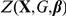 altogether here is to instead optimize the *pseudo-likelihood* [18] function for the above model, which has been previously shown to be asymptotically consistent and here yields fast parameter estimates. We define the negative log-pseudo-likelihood function as
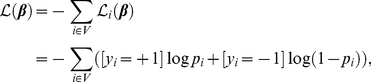
(2)where the conditional probability of individual 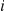
's phenotype given 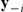 is denoted as 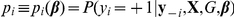. We note that evaluating and differentiating the pseudo-likelihood does not depend on the partition function, as under the above model, the conditional probability for individual 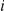 given all other individuals' phenotypes 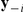 is given by
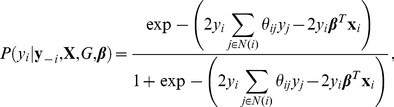
(3)where 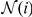 denotes the set of neighbors of individual 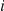 with respect to graph 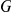 and we note that the partition function 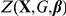 has dropped out. Thus, to perform genome-wide associations, we optimize the above function with respect to 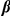: this can be done by using a gradient-based optimization whereby we iteratively update the vector of weights 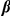 using the gradient of the pseudo-likelihood. The above pseudo-likelihood corresponds to solving a logistic regression problem with covariates 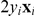 and an additive term for each individual 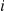, given by 
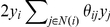
, which models the contribution of other individuals' phenotypes in determining the phenotype of 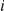. We remark that computing this additive term need only be done once and requires time that is quadratic in the number of individuals, which contrasts with cubic runtime required by LMM-based methods [12], [13]. Furthermore, the time required for parameter estimation per SNP is linear in the number of individuals, as we need only compute a conditional probability 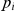 for each individual and corresponding derivatives with respect to weight vector 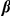. The resulting optimization problem is convex, with a unique global optimum, so we are guaranteed to obtain a unique solution 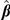 that maximizes the pseudo-likelihood, in contrast to parameter estimation in LMMs, which may be prone to local minima.

### Pseudo-likelihood estimation in the conditional random field

The gradient descent updates for parameter estimation under our method take the form 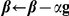, where 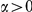 is a learning rate parameter and 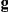 is the gradient of the pseudo-likelihood given by
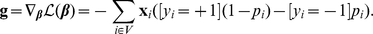
The weight vector 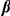 is updated until convergence in 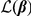. For our experiments, we used 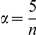, which was selected for fast convergence.

### Significance testing of SNPs

Given an estimate 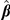 that minimizes the negative log-pseudo-likelihood function, define the robust variance estimator [19] as
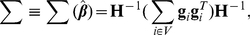
(4)where 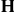 is the Hessian matrix of the pseudo-likelihood objective function, given by
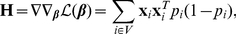
(5)and 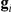 is given by
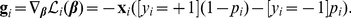
(6)The statistic 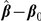 has been shown to be asymptotically distributed according to 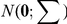 [20]–[23]. In particular, it follows that the statistic 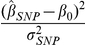 is 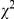 with one degree of freedom, where 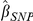 is the learned weight for a given SNP, 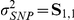 and 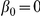 is the weight for the SNP under the null hypothesis. The above is equivalent to performing a Wald test on 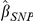 with a robust variance estimator for the variance of 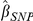.

### Measuring genomic inflation

Given 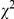 statistics 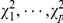 for each SNP 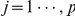 of interest, we can compute a genomic inflation factor 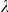 [2] as
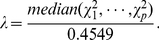
(7)


### Evaluating model performance

To gauge the calibration and discrimination of our model for both weaker and stronger associations, we generated data with different SNP regression weights using a GLMM. For each SNP, we generated SNP-phenotype associations by setting the SNP regression weight 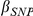 to 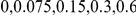, sampling a vector 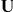 from a Gaussian distribution 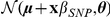 and finally generating the output phenotype for each individual with probability 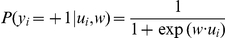, where 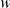 was chosen in order to obtain synthetic phenotype data with similar phenotype frequencies as those of real phenotype data.

Given a set of model p-values for synthetic data, we define a false positive (FP) to be a SNP that has a significant p-value for some significance level 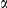 for synthetic data in which 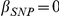. A true negative is defined as a SNP that is not significant at significance level 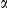 for synthetic data in which 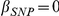. True positives (TP) and false negatives (TN) are defined similarly for synthetic data with 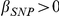. By varying the significance level 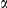, we can evaluate the performance of our model using a receiver operating characteristic (ROC) curve, or plotting true positive rate 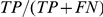 as a function of the false positive rate 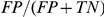 for various synthetic datasets with 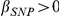. The performance can then be summarized using the area under the ROC curve, or AUC. Methods that have higher AUC have higher statistical power in discriminating between real and spurious associations.
